# Structure and conductivity enhanced treble-shelled porous silicon as an anode for high-performance lithium-ion batteries

**DOI:** 10.1039/c9ra06576h

**Published:** 2019-11-01

**Authors:** Yangfan Lin, Hanqing Lin, Jingwei Jiang, Deren Yang, Ning Du, Xueqin He, Jianguo Ren, Peng He, Chunlei Pang, Chengmao Xiao

**Affiliations:** State Key Lab of Silicon Materials, School of Materials Science and Engineering, Zhejiang University Hangzhou 310027 People's Republic of China dna1122@zju.edu.cn +86-571-87952322 +86-571-87953190; BTR New Energy Materials Inc Shenzhen 518106 P. R. China

## Abstract

Silicon is regarded as the next generation anode material for lithium-ion batteries because of its high specific capacity, low intercalation potential and abundant reserves. However, huge volume changes during the lithiation and delithiation processes and low electrical conductivity obstruct the practical applications of silicon anodes. In this study, a treble-shelled porous silicon (TS-P-Si) structure was synthesized *via* a three-step approach. The TS-P-Si anode delivered a capacity of 858.94 mA h g^−1^ and a capacity retention of 87.8% (753.99 mA h g^−1^) after being subjected to 400 cycles at a current density of 400 mA g^−1^. The good cycling performance was due to the unique structure of the inner silicon oxide layer, middle silver nano-particle layer and outer carbon layer, leading to a good conductivity and a decreased volume change of this silicon-based anode.

## Abstract 2

In this paper, a treble-shelled porous silicon structure is synthesized through three-step approach to enhance the structural stability and conductivity.

## Graphical abstract

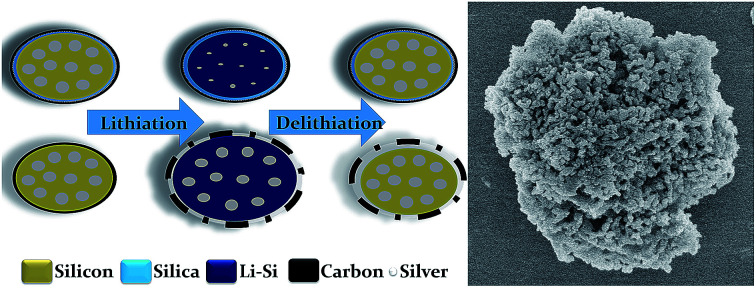

## 1. Introduction

Rapid development in the field of portable devices, electric vehicles and large-scale energy storage systems demands for high-performance lithium-ion batteries (LIBs) with high energy density, long cycle life, low fabrication cost and good security capability.^1–3^ Among all the promising next-generation anodes for LIBs, silicon (Si) exhibits the most competitive specific capacity of ∼4200 mA h g^−1^ (Li_22_Si_5_) and reliable safety performances (no lithium metal dendrite formation).^4,5^ However, its large volume expansion and contraction (∼300%) during lithiation and delithiation processes induce the pulverization of the Si structure, and in consequence leads to a poor cycling stability.^6^ Besides, the large volume change of Si particles increases the instability of the interface between the electrode and electrolyte, leading to the fracture and repetitive formation of a solid-electrolyte interphase (SEI) film.^7^ Various structures, such as nanoparticles,^8,9^ nanotubes,^10–12^ nanofibers,^13,14^ core–shell structures,^15,16^ yolk–shell structures,^17,18^ and porous structures,^19–22^ have been synthesized in an effort to buffer the volume expansion during the lithiation process. Among the above-mentioned structures, porous Si with a coating layer attracts considerable attention. Its inner porous structure has enough internal space to accommodate the volume expansion and its cover shell can limit the formation of SEI by avoiding the direct contact of Si and electrolytes.^23^

In the last decade, many attempts have been made to synthesize these Si/carbon porous structures to be used as anode materials in LIBs. Zhang *et al.* report a three-dimensional bicontinuous silicon anode formed by depositing a Si film on a porous nickel metal template *via* a chemical vapor deposition (CVD) method. This bicontinuous 3D porous Si anode exhibits an especially high reversible capacity of 2660 mA h g^−1^ after 100 cycles at a current density of 0.3 C.^24^ A. Magasinski *et al.* deposited Si nanoparticles on the surface of carbon black dendritic particles, which were subsequently assembled into a porous rigid spherical granule *via* CVD.^25^ The so-called C–Si nanocomposite spherical granules delivered a reversible specific capacity of 1590 mA h g^−1^ with almost no capacity fading after 100 cycles at a current density of 1 C. The porous Si structure synthesized by the CVD process delivered a good electrochemical performance from the standpoint of specific capacity and cycle life; however, the fabricating cost of a porous Si structure by CVD is an insurmountable challenge for commercial applications. Recently, our group demonstrated a large-scale, low-cost and convenient preparation method of Si@C three-dimensional porous structures *via* simple annealing and acid pickling processes of home-build magnesium silicide (Mg_2_Si, ∼5$ per kg).^26^ These Si@C three-dimensional porous structures can accommodate large volume expansion during the lithiation process and deliver a high reversible capacity of ∼1700 mA h g^−1^ after 70 cycles. The production technology of porous Si from Mg_2_Si has a good prospect for mass production; however, the as-synthesized porous Si is subject to structural instability during the lithiation and delithiation processes. In addition, the low conductivity of porous Si is an obstruction for the application of porous Si synthesized from Mg_2_Si.

Herein, treble shells constituted by an inner silicon oxide layer, a silver (Ag) nanoparticle interlayer and an outermost carbon layer are compounded on the surface of porous silicon. TS-P-Si electrodes exhibit remarkable cycling stability, which can be attributed to the synergistic effect of treble shells, which limit the expansion of silicon during cycling,^27,28^ improve the electrical conductivity of electrodes^29^ and eliminate the active materials from electrolytes to prevent unwanted reactions.^30^

## 2. Experimental section

### 2.1. Materials

Mg_2_Si (purity, >97%) was synthesized through a home-built continuous preparation apparatus using Si (purity, >98%) and Mg (purity, >99%) as the sources. (3-Mercaptopropyl)triethoxysilane (MPTS, GC, >96%, Aladdin, China), HCl (AR, 36%–38%, Sinopharm Chemical Reagent Co., Ltd., China), H_2_SO_4_ (AR, 98%, Sinopharm Chemical Reagent Co., Ltd., China), H_2_O_2_ (AR, 30%, Sinopharm Chemical Reagent Co., Ltd., China), ammonium hydroxide (AR, 25–28%, Sinopharm Chemical Reagent Co., Ltd., China), polyvinylpyrrolidone (40 000 wt, Sigma-Aldrich, America), ethylene glycol (GC, >99.5%, Aladdin, China), *n*-octylamine (GC, >99.5%, Aladdin, China), AgNO_3_ (>99%, Sigma-Aldrich, America), super P carbon black (CP, Sinopharm Chemical Reagent Co., Ltd., China), sodium carboxymethyl cellulose (∼90 000, Sigma-Aldrich, America), and electrolyte solutions (Dongguan shanshan battery materials Co., Ltd, China) were used.

### 2.2. Synthesis of TS-P-Si

TS-P-Si was synthesized *via* a three-step approach. First, 5 g Mg_2_Si powder was thermally treated in air atmosphere at a temperature of 80 °C for 1 h and then the powder was washed using diluted hydrochloric acid (0.2 mol L^−1^) to remove MgO and any residual Mg_2_Si. Porous silicon was collected as a precursor after vacuum drying at the temperature of 80 °C. Second, 500 mg of the precursor porous silicon particles were oxidized with a 40 mL solution H_2_SO_4_ : H_2_O_2_ = 1 : 1 in volume for 10 min to form a silicon oxide (SiO_*x*_) layer on the surface and plenty of hydroxyls were introduced on the surface of the silicon layer. Third, 100 μL MPTS and 60 μL aqueous ammonium hydroxide (25–28%) were mixed in 20 mL ethanol, then 200 mg oxidized porous silicon (O-P-Si) particles were dispersed in the above solution and stirred for 12 h at room temperature. The resulting MPTS-connected O-P-Si particles were centrifuged and washed with ethanol for several times and dried at 80 °C. The MPTS-connected O-P-Si particles (60 mg) were dispersed in 75 mL ethylene glycol with 5 mg PVP, then 25 mL AgNO_3_ solution was added to the MPTS-connected O-P-Si particles solution to obtain a final AgNO_3_ concentration of 3.5 mmol L^−1^, and thoroughly mixed. Octylamine (100 μL) was rapidly added in the MPTS-connected O-P-Si and AgNO_3_ mixed solution, and then the solution was stirred for 1 h. The Ag-coated porous silicon (P-Si/Ag) particles were collected after centrifugation, washed and dried. Finally, the P-Si/Ag particles were coated with a carbon layer *via* thermal decomposition of acetylene gas at a temperature of 800 °C for 1 h.

### 2.3. Characterization

The morphology of the as-prepared samples was characterized *via* field emission scanning electron microscopy (FESEM, HITACH S4800) and transmission electron microscopy (TEM, PHILIPS F200). Energy dispersive spectroscopy (EDS) mapping images and line scanning images were recorded by a FEI Titan ChemiS TEM equipped with a probe-corrector and a Super-X EDS detector system. X-ray photoelectron spectroscopy (XPS) was performed by employing a Thermo ESCALAB 250Xi spectrometer with a monochromatic Al Kα line (1486.6 eV). The crystal structures of the materials were determined using a high power X-ray diffractometer (XRD) on a Rigaku D/max-ga X-ray diffractometer, where the Cu Kα radiation was 1.54 Å.

### 2.4. Electrochemical characterization

The 2025-type cell, composed of TS-P-Si as the working electrode and lithium metal as the counter electrode, was encapsulated in a glove box (Mbraun, labstar, Germany) under argon atmosphere. The slurry casting on the Cu collector was constituted of active materials (TS-P-Si, P-Si/Ag and O-P-Si), sodium carboxymethyl cellulose (CMC) and super P carbon black, in a mass ratio of 7 : 2 : 1. The loading of active materials on the electrode was 2.4 mg cm^−2^. The electrolyte solution consisted of LiPF_6_ in dimethyl carbonate (DMC) and ethylene carbonate (EC) (1 : 1 volume ratio) with 5 vol% fluoroethylene carbonate (FEC) as an additive. The cyclic voltammetry (CV) curve was recorded on a CHI660D system at a scan rate of 0.1 mV s^−1^. The Nyquist plot was acquired on the CHI660D system in the frequency range from 100 kHz to 100 mHz. The galvanostatic discharge–charge data was collected using a Neware CT-4008-5V50 mA system in the potential range of 0.001–1.2 V.

## 3. Results and discussion

**Scheme 1:**
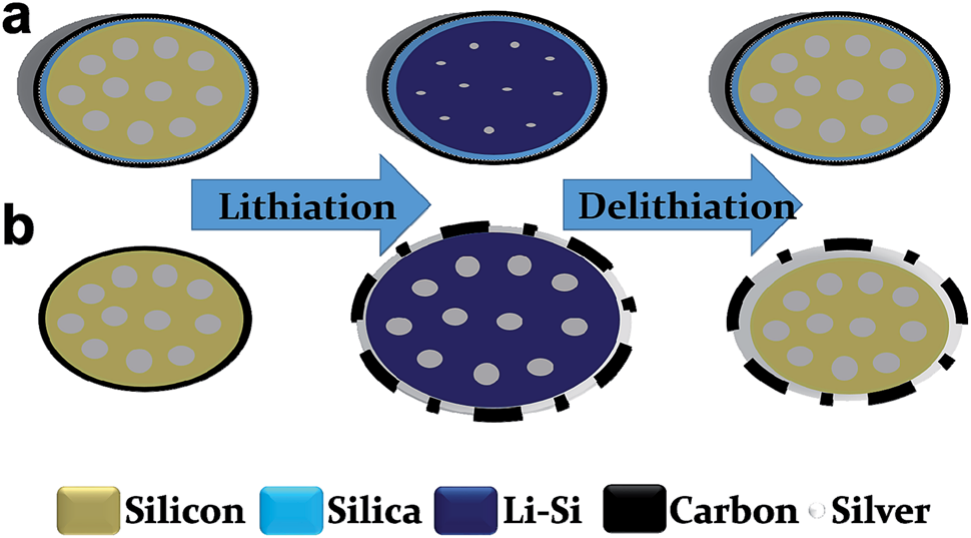
Schematic of (a) TS-P-Si and (b) P-Si@C during lithiation and delithiation processes.

Scheme 1 shows a representation of the structural transformations of TS-P-Si (a) and bare carbon-shelled porous Si (b) during the lithiation/delithiation process. As shown in Scheme 1a, the robust treble-shelled system constituted of a silicon oxide layer, a Ag nanoparticle layer and a carbon layer, in which the silicon oxide layer has a strong constraining effect and limits the outward expansion tendency of the inner Si. Consequently, Si tends to expand inward to fill its internal porosity during the lithiation process. Due to abundant voids in the porous Si that can accommodate the expansion of Si, the whole TS-P-Si structure will remain stable and even afford no volume change during the lithiation and delithiation processes. Therefore, the silica layer can maintain structural integrity and the outmost carbon layer can ensure the isolation of the internal active material from electrolytes to prevent side reactions after long cycles.^31^ However, for the carbon-shelled porous silicon, as shown in Scheme 1b, the carbon layer will be destroyed without a structural support, and the freshly exposed surface of active materials will produce side reactions with the electrolyte solution and eventually lead to the repeated regeneration of the SEI film and cause electrical isolation between the active materials.

**Fig. 1:**
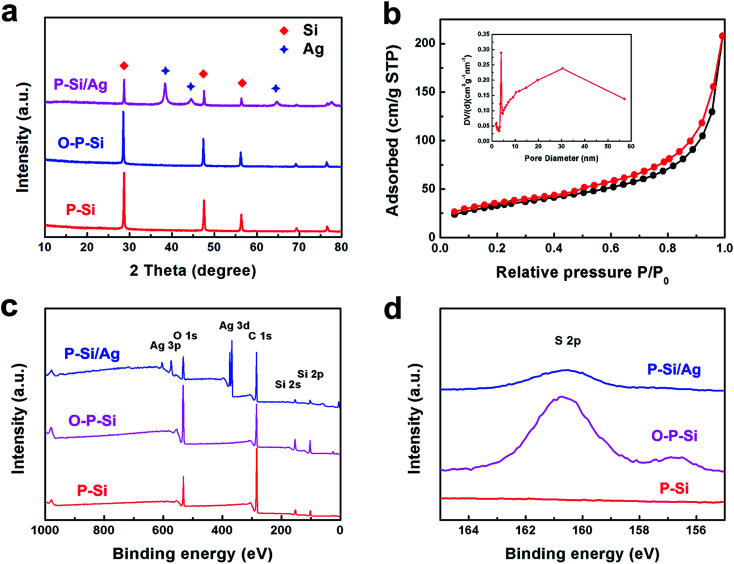
(a) XRD patterns of P-Si, MPTS-connected O-P-Si and P-Si/Ag; (b) N_2_ adsorption/desorption isotherms of TS-P-Si; insert is the pore size distribution curve of TS-P-Si; (c) XPS spectra of P-Si, MPTS-connected O-P-Si and P-Si/Ag; (d) S 2p XPS spectra of P-Si, MPTS-connected O-P-Si and P-Si/Ag.

TS-P-Si is synthesized *via* a three-step approach: first, porous Si was oxidized with a mixture of concentrated sulfuric acid and hydrogen peroxide in a ratio of 1 : 1 in volume for 10 min, to form a silicon oxide layer on the surface; second, Ag nanoparticles are deposited on the surface of the silicon oxide layer through a Ag mirror reaction;^32^ third, the outmost carbon layer is obtained *via* acetylene thermal decomposition. Fig. 1a shows the XRD patterns of the products along the synthetic process. As can be seen, P-Si/Ag exhibits the typical {1 1 1}, {2 2 0}, {3 1 1} planes of crystalline Si (JCPDS no. 27-1402) and {1 1 1}, {2 0 0}, {2 2 0} planes of crystalline Ag (JCPDS no. 04-0783). In addition, no distinctive amorphous phase distinguished from the XRD patterns of O-P-Si and P-Si/Ag indicates that the thickness of the silicon oxide layer is low. Therefore, the silicon oxide layer will just consume a limited amount of lithium and has no significant influence on the first coulombic efficiency of the TS-P-Si anodes. The average size of Ag particles calculated *via* the Scherrer equation is 16.9 nm. BET analysis was conducted to estimate the porosity of TS-P-Si after the carbon coating process. As shown in Fig. 1b, TS-P-Si has a BET surface area of 117.05 m^2^ g^−1^ with an average Barrett–Joyner–Halenda (BJH) pore diameter of 12.4 nm, indicating that the porous structure still remained unchanged after the deposition of the carbon shell. Furthermore, XPS was employed to analyze the surface state transformation during the synthetic process of the Ag nanoparticle layer. In order to deposit the Ag nanoparticles on the surface of porous Si, a hydroxysulphonyl group was introduced on the surface of porous Si first *via* the coupling effect of hydroxyl groups on the surface of the silicon oxide layer and (3-mercaptopropyl) triethoxysilane. After hydroxysulphonyls collect Ag ions on the surface of porous Si, an Ag nanoparticle layer could be synthesized after the reduction of the Ag ions by *n*-octylamine.^33^Fig. 1c shows the XPS spectra of O-P-Si, MPTS-connected O-P-Si and P-Si/Ag, indicating that the Ag nanoparticles have been deposited on the surface. Fig. 1d shows the S 2p XPS spectra of P-Si, MPTS-connected O-P-Si and P-Si–Ag. As can be seen, the sulfur element appears on the surface of O-P-Si particles after MPTS treatment and decreases after Ag nanoparticle deposition, which can demonstrate that the sulfhydryl moiety is connected on the surface of O-P-Si nanoparticles and consumed during the Ag nanoparticle deposition process.

**Fig. 2:**
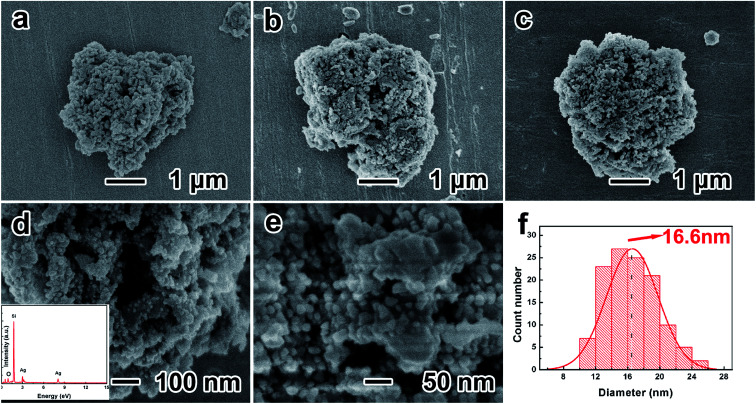
Low-resolution SEM images of (a) P-Si, (b) MPTS-connected O-P-Si, (c) P-Si/Ag; (d) high-resolution SEM image of P-Si/Ag; (e) high-resolution SEM image of Ag nanoparticles on the surface of porous silicon; (f) diameter distribution diagram of Ag nanoparticles on the surface of the P-Si/Ag structure.

Fig. 2a–c show the low-resolution SEM images of porous silicon, O-P-Si and P-Si/Ag, suggesting that the porous structure has a diameter of ∼3 μm. Porous silicon is constituted primarily by Si nanoparticles, and the space between particles composes the porous structure. After the oxidation process, the silicon oxide layer connects the adjacent nanoparticles on their surfaces and the resulting O-P-Si has higher structural integrity. Fig. 2c shows the morphology of the uniform deposition of a Ag layer on the surface of porous silicon. The high-resolution SEM image of P-Si/Ag demonstrates that the Ag nanoparticles are compactly attached on the surface of porous silicon (Fig. 2d). The EDS spectra in the inset of Fig. 2d show that the elemental composition of Si, Ag and O are 59.36%, 21.34% and 19.31%, respectively. Fig. 2f shows the diameter statistical distribution diagram for Ag nanoparticles on the surface of the P-Si/Ag from Fig. 2e. It can be seen that the average diameter of Ag nanoparticles is ∼16.6 nm, which corresponds to the calculated result *via* the Scherrer equation.

**Fig. 3:**
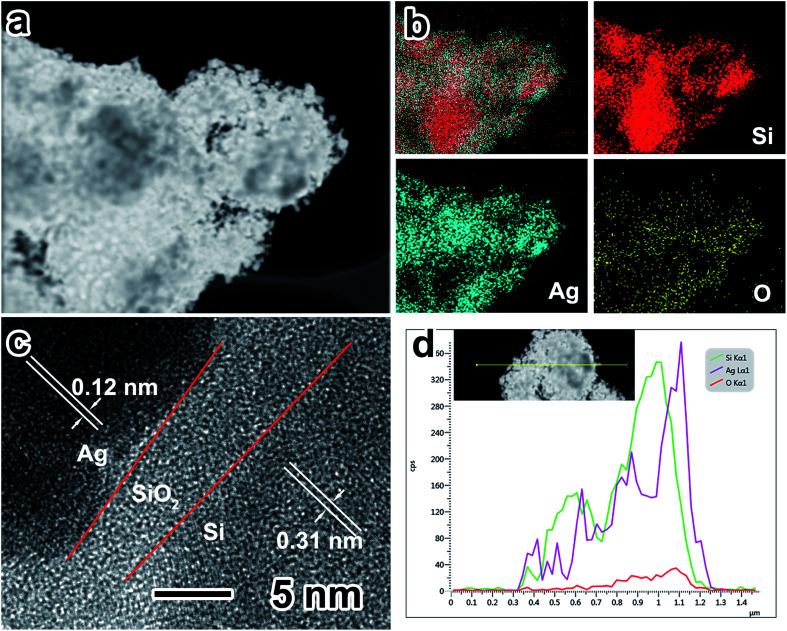
(a and b) EDX elemental mapping of P-Si/Ag; (c) high-magnification TEM image of P-Si–Ag; (d) EDX elemental line scanning of P-Si/Ag.

The EDS elemental mapping of Ag nanoparticle-coated porous Si (Fig. 3a and b) was conducted to estimate the distribution of major elements (Si, Ag and O). It can be noticed that Si is distributed in the whole porous structure and elements such as Ag and O are distributed only on the surface of porous Si. Fig. 3c shows the HRTEM image of the sandwich construction contributed by inner Si, the middle silicon oxide layer and outer Ag nanoparticles. The lattice distances of ∼0.31 and ∼0.12 nm correspond to the {1 1 1} face of Si and the {2 0 2} face of Ag, respectively. Between the inner Si and Ag nanoparticle layer, the silicon oxide layer has a thickness of ∼5 nm. It should be mentioned that an appropriate thickness of the silicon oxide layer is vital for the electrochemical performance of the Si anode.^34^ The silicon oxide layer can maintain the structure stability of porous Si during multiple cycles; however, a thick silicon oxide layer will consume too much of the lithium source, resulting in the lowering of the initial coulombic efficiency and a poor electrical conductivity. In addition, the EDS elemental line scanning of Ag nanoparticle-coated porous Si (Fig. 3d) demonstrates that the elements of Ag and O are uniformly distributed on the surface of Si.

**Fig. 4:**
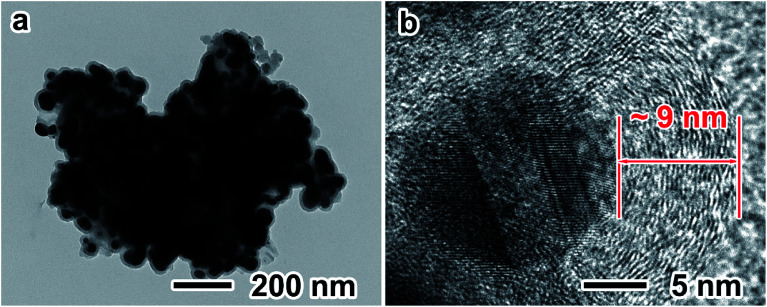
(a) Low-magnification TEM image of TS-P-Si; (b) high-magnification TEM image of TS-P-Si.

TS-P-Si was synthesized after carbon coating through acetylene thermal decomposition. Fig. 4 shows the TEM images of TS-P-Si. It can be seen that a uniform carbon layer covers the whole porous structure, as shown in Fig. 4a. The high-magnification TEM image in Fig. 4b shows that the thickness of the carbon layer is ∼9 nm.

**Fig. 5:**
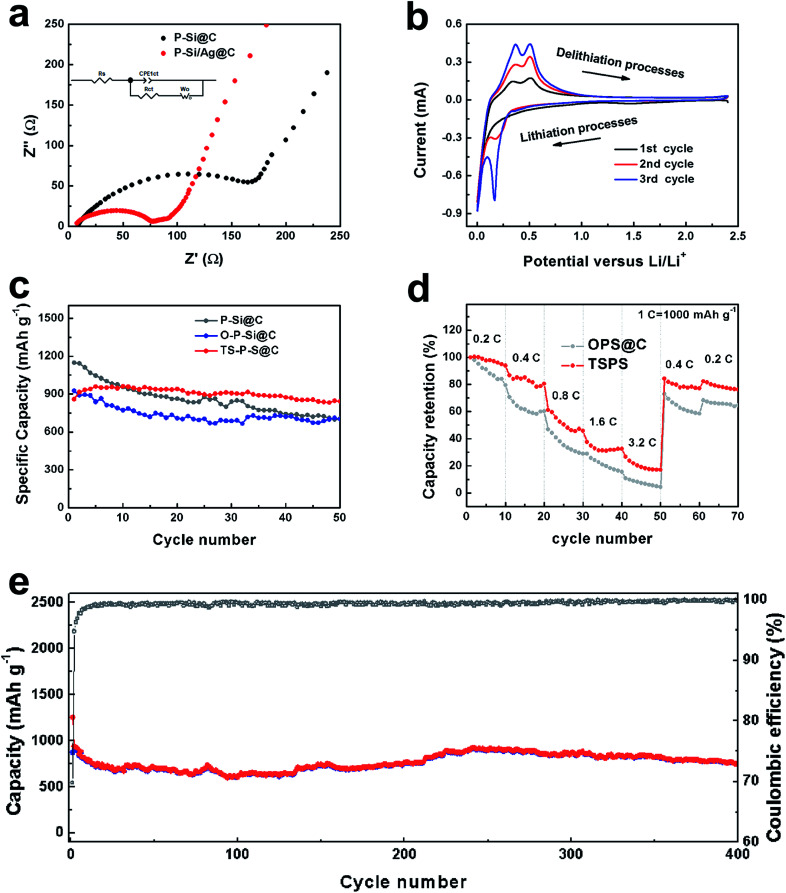
(a) Nyquist plots of P-Si@C and TS-P-Si obtained by applying a sing wave over the frequency range from 100 kHz to 0.01 Hz. (b) Cyclic curves for TS-P-Si electrodes between 2.5 V and 0.01 V *versus* Li/Li^+^ at the scan rate of 0.1 mV s^−1^. (c) Cycling performance of the P-Si@C electrodes and TS-P-Si electrodes at the same current density of 400 mA g^−1^; (d) rate performance of the P-Si@C electrodes and TS-P-Si electrodes. (e) Long time cycling performance of the TS-P-Si electrodes at the current density of 400 mA g^−1^.

To further corroborate the stability of the TS-P-Si structure during lithiation and delithiation processes, the TS-P-Si particles were used to prepare an anode, and then evaluated their electrochemical performance in LIBs. Fig. 5a shows the Nyquist plots of bare carbon-coated porous silicon (P-Si@C) and TS-P-Si (before a cycle) obtained by applying a sinusoidal current over the frequency range of 100 kHz–0.01 Hz. The illustration insertion shows the equivalent circuit of the EIS impedance simulation. *R*_s_ represents the internal impedance of the tested LIBs, while *R*_ct_ and CPEct correspond to the charge-transfer resistance and the constant phase element of the interface between electrode and electrolyte, respectively. *W*_o_ is associated with the Warburg impedance, corresponding to the Li-ion diffusion process.^35^ As shown in Fig. 5a, the semicircle on the medium-frequency region corresponds to the *R*_ct_ and CPEct of the electrode/electrolyte interface, and the sloping line in the low-frequency region corresponds to the lithium-ion diffusion process within the electrode materials. The charge-transfer differences of P-Si@C and TS-P-Si were investigated by further modeling AC impedance spectra based on a modified equivalent circuit. The fitted *R*_ct_ quantitative values for P-Si@C and TS-P-Si are 176.4 and 72.9 Ω, respectively, indicating that the Ag nanoparticle layer can act as a current collector network to improve the electrical conductivity and accelerate the electron transport during the lithiation/delithiation processes, resulting in a significant improvement in the electrochemical performance of the TS-P-Si anode.

Fig. 5b shows the typical cyclic voltammetry (CV) curve of an anode prepared by TS-P-Si, tested in the range 0.01–2.5 V *versus* Li/Li^+^ at a scanning rate of 0.1 mV s^−1^. In the first cycle, two cathodic peaks located at 0.01 V, and two anodic peaks located at 0.38 and 0.51 V can be observed, corresponding to the reversible lithiation/delithiation process of Si.^36^ In the subsequent two cycles, the current intensity of the redox peaks increases gradually and the location of redox peaks remains at the same potential value, indicating an increased speed of the lithiation/delithiation reaction.^37^Fig. 5c shows a comparison of the electrochemical performances of P-Si@C, carbon-coated oxidized porous silicon (O-P-Si@C) and treble-shelled porous silicon (TS-P-Si). Table 1 counts the electrochemical performances of the above materials after 50 cycles. O-P-Si@C electrodes, with a 75.80% capacity retention, show a much better cycling performance than P-Si@C (with only 61.48% capacity retention). This can be attributed to the stable structure of the silicon oxide layer, which can buffer the volume expansion of the inner silicon structure during cycles. As a result, the O-P-Si@C structures can maintain the stability of the SEI and deliver stable cycling performance. Furthermore, the best electrochemical performance of the TS-P-Si electrodes can be attributed to the excellent electrical conductivity of Ag, which can be seen in the EIS test in Fig. 5a. Fig. 5d shows the rate performance of the O-P-Si@C and TS-P-Si electrodes. The O-P-Si@C shows 84.02%, 60.35%, 28.84%, 15.63%, 4.48%, 58.94%, 64.53% capacity retentions at the current densities of 0.2C, 0.4C, 0.8C, 1.6C, 3.2C and then back to 0.4C and 0.2C, respectively. For comparison, TS-P-Si shows 93.84%, 80.52%, 45.82%, 32.55%, 17.13%, 77.17%, 76.34% capacity retentions under the similar conditions. Eventually, TS-P-Si was subjected to tests on cycling stability over multiple cycles. As shown in Fig. 5e, TS-P-Si preserves about 753.99 mA h g^−1^ charge capacity (∼87.8% capacity retention to first charge) after 400 cycles, showing particularly good cycling stability.

**Table 1:** The comparison of the electrochemical performance of P-Si@C, O-P-Si@C and TS-P-Si before and after 50 cycles

	1st Capacity (mA h g^−1^)	50th Capacity (mA h g^−1^)	Capacity retention (%)
P-Si@C	1148.06	705.80	61.48
O-P-Si@C	925.28	701.40	75.80
TS-P-Si	858.94	841.64	97.99

**Fig. 6:**
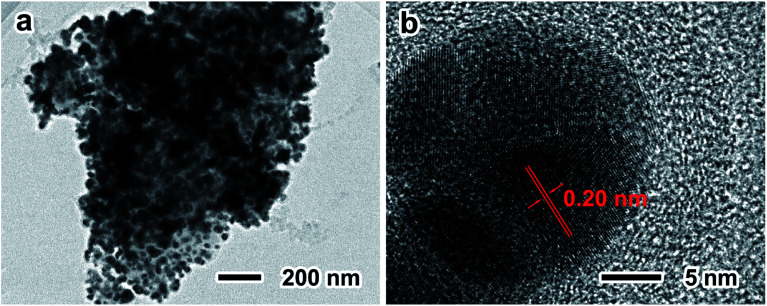
Low- (a) and high-magnification TEM (b) images of TS-P-Si after 50 cycle times at a current density of 400 mA h g^−1^.

During the lithiation and delithiation processes, the silicon oxide layer and the carbon layer can maintain the structure stability of TS-P-Si. In addition, the Ag nanoparticle layer and carbon layer can maintain the electrical conductivity of the treble-shelled porous structure. The synergistic effects of treble shells contribute to the especially stable electrochemical performance of the TS-P-Si anode. Fig. 6 shows the TEM images of TS-P-Si after 50th cycle at a current density of 400 mA h g^−1^. As can be seen, the integrity of treble-shelled porous structures is maintained after 50th cycle (Fig. 6a), and the Ag nanoparticles remain in their initial phase without reacting with lithium ions, as the lattice distance of ∼0.20 nm is definitely identified as the {103} face of Ag (Fig. 6b); and always act as a current collector on the surface of TS-P-Si during the charge/discharge process. TS-P-Si has a stable structure that will not be destructed by the stress induced by the volume expansion/shrinkage of inner silicon during the cycling process. Simultaneously, TS-P-Si can maintain the intact conductive network consisting of Ag nanoparticles and a carbon layer in the protection of silicon oxide layer. As a consequence, the TS-P-Si delivers an excellent electrochemical performance as anode materials for LIBs.

## 4. Conclusion

In summary, a novel treble-shelled porous silicon structure with structural-enhanced and electrical conductivity-enhanced layers was synthesized *via* a three-step approach. Due to the protection ability of an inner silicon oxide layer and an outer carbon layer, TS-P-Si can maintain structural stability during lithiation and delithiation processes. Furthermore, the middle Ag nanoparticle layer and outer carbon layer can improve the electrical conductivity of the integral structure of porous silicon. Therefore, the treble-shelled porous silicon anode delivered an ultra-stable cycling life (86.14% capacity retention after 400 cycles at a current density of 400 mA g^−1^) as an anode material for LIBs.

## Conflicts of interest

There are no conflicts to declare.

## Supplementary Material
